# Over eight years of transfusion independence with continuous erythropoietin receptor activator and Roxadustat in transfusion-dependent low-risk myelodysplastic syndrome

**DOI:** 10.1093/omcr/omaf092

**Published:** 2025-07-14

**Authors:** Tatsuyoshi Ikenoue, Yoshiyuki Furumatsu, Tetsuya Kitamura

**Affiliations:** Data Science and AI Innovation Research Promotion Center, Shiga University, 1-1-1 Baba, Hikone, Shiga 522-8522, JAPAN; Nephrology, Hirano Keijin Clinic, 3-2-20 Nagayoshi-Nagahara-Higashi, Hirano, Osaka 547-0013, JAPAN; Nephrology, Hirano Keijin Clinic, 3-2-20 Nagayoshi-Nagahara-Higashi, Hirano, Osaka 547-0013, JAPAN

**Keywords:** myelodysplastic syndrome, Epoetin Beta Pegol, transfusion independence, Hemodialysis, safety, hypoxia-inducible factor

## Abstract

We report a case of a 65-year-old Japanese woman with low-risk myelodysplastic syndrome (MDS) on hemodialysis who achieved transfusion independence for over eight years with combined epoetin beta pegol (continuous erythropoietin receptor activator, CERA) and roxadustat. Transfusion-dependent since 2008, she showed a temporary response to darbepoetin and CERA, initiated in August 2016. Roxadustat was added in January 2020, leading to sustained transfusion independence. No serious adverse events, such as progression to acute leukemia or clinically significant thyroid dysfunction, were observed during this period.

## Introduction

Myelodysplastic syndrome (MDS) refers to clonal hematopoietic stem cell disorders characterized by ineffective hematopoiesis, peripheral blood cytopenia, and risk of progression to acute myeloid leukemia [1]. Anemia, the most common type of cytopenia in MDS, significantly impacts quality of life [2]. For low-risk MDS, erythropoiesis-stimulating agents (ESAs) are the first-line treatment; however, some patients are refractory or lose their response to ESAs over time [3].

Roxadustat, a hypoxia-inducible factor prolyl hydroxylase (HIF-PH) inhibitor, promotes erythropoiesis by increasing endogenous erythropoietin levels and improving iron utilization [4]. It has been approved for the treatment of anemia in chronic kidney disease (CKD), including in patients on dialysis [5].

We have previously reported earlier phases of this patient’s treatment course [6, 7]. The current report presents important new findings, including long-term follow-up data through September 2024, demonstrating sustained efficacy and safety over an extended period.

## Case presentation

A Japanese woman was diagnosed with MDS in 2008. Bone marrow examination revealed 40%–50% cellularity with trilineage dysplasia and 2% blasts. The diagnosis was based on cytological bone marrow analysis and peripheral blood counts, showing mild dysplasia in the erythroid lineage (anisocytosis, multinucleated erythroblasts) and pseudo-Pelger-Huët anomaly in the granulocytic lineage. Karyotype analysis demonstrated a normal karyotype, and all MDS-related FISH tests were negative. Next-generation sequencing was not performed. She had refractory anemia, categorized as low-risk (IPSS low or Int-1).

**Figure 1:**
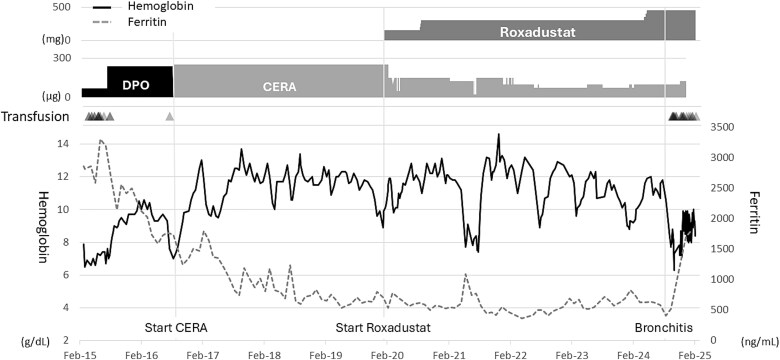
Trends in hemoglobin and ferritin levels and reticulocyte count, along with transfusion and treatment history. Key dates: CERA initiation (august 16, 2016), roxadustat initiation (January 9, 2020), and transfusion resumption (September 10, 2024). Black triangles, blood transfusions. Solid black line, hemoglobin (g/dL, left axis). Dashed gray line, ferritin (ng/mL, right axis). Upper panels show medication dosages: Roxadustat (mg); DPO and CERA (μg).

She had diabetes since 1991, and the resultant renal insufficiency led to hemodialysis initiation in January 2015. Before dialysis, she required weekly transfusions for 44 weeks (Fig. 1), resulting in congestive heart failure, secondary hemochromatosis, and a diagnosis of transfusion-associated circulatory overload (TACO). In March 2015, epoetin alfa plus darbepoetin (DPO) was initiated alongside hemodialysis. In July 2015, DPO was increased to 240 μg/week, resolving her anemia. However, resistance to DPO began to develop in March 2016, and by July 2016, transfusions were resumed.

On August 16, 2016, the ESA was switched to continuous erythropoietin receptor activator (CERA, 250 μg/week). Her hemoglobin gradually increased, leading to transfusion independence for over three years (Fig. 1). In December 2019, despite stable iron and unchanged treatment, her hemoglobin decreased, reaching 8.9 g/dL in January 2020. On January 9, 2020, roxadustat (150 mg/week, later increased to 300 mg/week) was added to CERA (150 μg/week). Her hemoglobin increased, and a switch to roxadustat monotherapy was attempted by reducing CERA to 50 μg/week. However, her hemoglobin then decreased, so both CERA (150 μg/week) and roxadustat (300 mg/week) were continued.

Since August 16, 2016, when the patient was 65 years old, she maintained transfusion independence (Fig. 1). However, beginning on July 8, 2024, she developed severe cough and nasal discharge, suggestive of acute bronchitis. Multiple tests for COVID-19 and influenza were negative. Treatments included inhaled corticosteroids and minocycline hydrochloride (200 mg/day). Despite treatment, the symptoms persisted for approximately 1.5 months. Subsequently, on September 10, 2024, transfusion was reintroduced due to a decrease in hemoglobin to 8.9 g/dL. No serious adverse events, such as progression to acute leukemia, were observed during the > 4 years of roxadustat use. Thyroid ultrasonography revealed no goiter. Informed consent was obtained from the patient for publication.

## Discussion

This report describes a patient with low-risk MDS on dialysis who achieved over eight years of transfusion independence (August 16, 2016, to September 9, 2024) with CERA and roxadustat combination therapy. This case provides evidence that such combination can achieve sustained transfusion independence and long-term treatment safety.

It is important to acknowledge that the patient’s anemia had dual etiologies—MDS and renal failure—making it challenging to precisely attribute the clinical response to either therapy alone. Nevertheless, the temporal relationship between treatment adjustments and hemoglobin responses suggests a true therapeutic effect.

Roxadustat activates the HIF pathway, promoting endogenous erythropoietin production and improving iron utilization [5]. In this case, sustained hematopoietic stimulation by CERA, coupled with increased endogenous erythropoietin via the HIF pathway and improved iron utilization by roxadustat, likely contributed to transfusion independence. Since the patient was on dialysis, we presume that roxadustat resulted in significant extrarenal erythropoietin production and improved iron utilization. Early clinical evidence demonstrated transfusion independence in 37.5% of patients with lower-risk MDS, with the highest response rates observed at the 2.5 mg/kg dose [8]. The subsequent MATTERHORN trial reported statistically significant benefits in patients with higher transfusion burden (36.1% vs. 11.5%, *P* = 0.047) [9]. Although these studies did not specifically target patients on dialysis or using CERA, they included patients with transfusion-dependent, lower-risk MDS; these findings suggest roxadustat’s potential efficacy, particularly in patients with higher transfusion needs.

Roxadustat’s long-term safety requires consideration. Although no serious adverse events were observed here during > 4 years of roxadustat use, excessive HIF pathway activation could theoretically promote MDS progression to leukemia. In the MATTERHORN Phase III trial, 3 out of 82 patients (3.7%) with lower-risk MDS treated with roxadustat progressed to acute myeloid leukemia, while no progression occurred in the placebo group [9]. Although this difference was not statistically analyzed, it warrants attention in clinical practice. Notably, our patient showed no signs of leukemic progression despite extended roxadustat use, suggesting that individual patient factors and close monitoring may mitigate potential risks. Roxadustat affects thyroid function via the HIF pathway, potentially causing hypothyroidism and low TSH [10]. In this case, regular thyroid function tests (TSH, FT3, and FT4) showed no abnormalities and no symptoms or signs of thyroid dysfunction, consistent with the ultrasound findings. This combination therapy may therefore be a useful treatment option for anemia in similar patients.

## Conclusion

This case suggests that CERA and roxadustat combination therapy may offer a safe and effective long-term treatment for achieving transfusion independence in selected patients with transfusion-dependent low-risk MDS, particularly those with concurrent renal anemia. The sustained transfusion independence observed over > 8 years provides valuable insights into both efficacy and safety. However, given the multifactorial nature of the patient’s anemia and the limitations of a single case report, these findings should be cautiously interpreted. Further research is needed to confirm these findings and optimize the treatment.

## References

[1] Greenberg PL, Stone RM, Al-Kali A. et al. Myelodysplastic syndromes, version 2.2017, NCCN clinical practice guidelines in oncology. J Natl Compr Cancer Netw 2017;15:60–87. 10.6004/jnccn.2017.0007 28040720

[2] Cazzola M, Malcovati L. Myelodysplastic syndromes--coping with ineffective hematopoiesis. N Engl J Med 2005;352:536–8. 10.1056/NEJMp048266 15703418

[3] Platzbecker U, Symeonidis A, Oliva EN. et al. A phase 3 randomized placebo-controlled trial of darbepoetin alfa in patients with anemia and lower-risk myelodysplastic syndromes. Leukemia 2017;31:1944–50. 10.1038/leu.2017.192 28626220 PMC5596208

[4] Haase VH . Mechanisms of hypoxia responses in renal tissue. J Am Soc Nephrol 2013;24:537–41. 10.1681/ASN.2012080855 23334390

[5] Fishbane S, Pollock CA, El-Shahawy M. et al. Roxadustat versus epoetin alfa for treating anemia in patients with chronic kidney disease on dialysis: results from the randomized phase 3 Rockies study. J Am Soc Nephrol 2022;33:850–66. 10.1681/ASN.2020111638 35361724 PMC8970450

[6] Ikenoue T, Naito H, Kitamura T. et al. Epoetin β pegol (continuous erythropoietin receptor activator, CERA) is another choice for the treatment of anemia in myelodysplastic syndrome: a case report. J Med Case Rep 2017;11:296. 10.1186/s13256-017-1468-z 29047386 PMC5648440

[7] Ikenoue T, Furumatsu Y, Kitamura T. Transfusion-dependent anaemia treatment using continuous erythropoietin receptor activator (epoetin β pegol) and roxadustat after darbepoetin treatment failure in low-risk myelodysplastic syndrome: a case report. Oxf Med Case Rep 2021;2021, 2021:omab026. 10.1093/omcr/omab026 PMC8143669 34055362

[8] Henry DH, Glaspy J, Harrup R. et al. Roxadustat for the treatment of anemia in patients with lower-risk myelodysplastic syndrome: open-label, dose-selection, lead-in stage of a phase 3 study. Am J Hematol 2022;97:174–84. 10.1002/ajh.26397 34724251

[9] Mittelman M, Henry DH, Glaspy JA. et al. Roxadustat versus placebo for patients with lower-risk myelodysplastic syndrome: Matterhorn phase 3, double-blind, randomized controlled trial. Am J Hematol 2024;99:1778–89. 10.1002/ajh.27410 38884137

[10] Otsuka E, Kitamura M, Funakoshi S. et al. Roxadustat has risks of reversible central hypothyroidism in patients undergoing hemodialysis: a single-center retrospective cohort study. Ren Fail 2024;46:2410375. 10.1080/0886022X.2024.2410375 39378117 PMC11463015

